# Comparison of blood volume biofeedback hemodialysis and conventional hemodialysis on cardiovascular stability and blood pressure control in hemodialysis patients: a systematic review and meta-analysis of randomized controlled trials

**DOI:** 10.1007/s40620-023-01844-0

**Published:** 2024-03-26

**Authors:** Emanuele Mambelli, Fabio Grandi, Antonio Santoro

**Affiliations:** 1 Nephrology and Dialysis Unit, AUSL Romagna - Ospedale Infermi, Rimini, Italy; 2 Medica S.P.A., Medolla, Italy; 3 Nephrology School University of Bologna, Bologna, Italy

**Keywords:** Intradialytic hypotension, Hemodialysis, Biofeedback system, Blood volume

## Abstract

**Background:**

Despite the improvements in hemodialysis (HD) technology, 20–30% of sessions are still complicated by hypotension or hypotension-related symptoms. Biofeedback systems have proven to reduce the occurrence of such events, but no conclusive findings can lead to wider adoption of these systems. We conducted this systematic review and meta-analysis of randomized clinical trials to establish whether the use of blood volume tracking systems compared to conventional hemodialysis (C-HD) reduces the occurrence of intradialytic hypotension.

**Methods:**

The PRISMA guidelines were used to carry out this systematic review. Randomized clinical trials that evaluated the incidence of intradialytic hypotension during C-HD and blood volume tracking-HD were searched in the current literature. PROSPERO registration number: CRD42023426328.

**Results:**

Ninety-seven randomized clinical trials were retrieved. Nine studies, including 347 participants and 13,274 HD treatments were considered eligible for this systematic review. The results showed that the use of biofeedback systems reduces the risk of intradialytic hypotension (log odds ratio = 0.63, *p* = 0.03) in hypotension-prone patients (log odds ratio = 0.54, *p* = 0.04). When analysis was limited to fluid overloaded or hypertensive patients, it did not show the same effect (log odds ratio = 0.79, *p* = 0.38). No correlation was found in systolic blood pressure drop during dialysis and in post-dialysis blood pressure.

**Conclusions:**

The use of blood volume tracking systems may be effective in reducing the incidence of intradialytic hypotension and allowing for easier attainment of the patients’ ideal dry body weight. New studies to examine the long-term effects of the use of blood volume tracking systems on real hard endpoints are needed.

**Graphical abstract:**

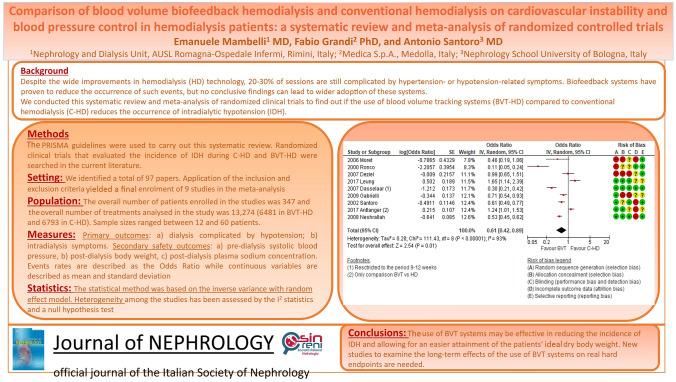

## Introduction

Hemodialysis (HD) is a life-saving therapy for end-stage kidney disease patients. Nevertheless, it is not a physiological treatment since it guarantees blood purification and water removal but does not achieve complete rehabilitation of the patient. Furthermore, hemodialysis leads to complications, among which intradialytic hypotension still represents the main one. This complication is even more evident because the mean age of the dialytic population is increasing, along with the increasing frequency of comorbidities such as diabetes and underlying cardiac disease [1].

Intradialytic hypotension occurs when the ultrafiltration rate is high, and it complicates 5 to 30% of all dialysis treatments depending on the definition used [2, 3].

When the rate of fluid removal by ultrafiltration is significantly faster than the refill rate of the intravascular space from the interstitial space, hypotension can occur because of intravascular volume depletion beyond the level at which blood pressure can be sustained by hemodynamic compensatory mechanisms. This is more common among patients with large interdialytic weight gain [4].

Many recent studies showed that intradialytic hypotension episodes and their frequency are related to increased mortality [4–8].

Over time, several strategies have been implemented to avoid hypotension episodes: a longer or more frequent dialysis regimen [9–15], diffusive-convective therapies [16], acetate-free biofiltration [17], accurate assessment of dry body weight by means of different tools and devices (bioimpedance, vena cava ultrasound, overhydration biomarkers, etc.) [18, 19], but none completely solved the problem. Daily dialysis or long duration dialysis might be the best solutions for optimal control of blood pressure during and between dialysis sessions [13–15]. Unfortunately, neither can be developed into an extensive form in routine practice.

Different systems have been suggested to prevent and avoid intradialytic hypotension in conventional hemodialysis (C-HD; three times per week, four hours per session) [20, 21]. These systems are based on the control of biological parameters (temperature, conductivity, blood volume reduction, blood pressure, etc.). Among these, blood volume tracking is a ‘biofeedback system’ that, via closed-loop control of blood volume variations, modifies the ultrafiltration rate and/or dialysate conductivity to maintain the blood volume stable and prevent blood volume from dropping below a defined threshold [20, 21]. It has been extensively studied by several authors, both in hypotension-prone patients as well as in overhydrated and hypertensive patients. Indeed, to date, it is uncertain whether blood volume tracking-HD is effective in reducing the occurrence of intradialytic hypotension compared to C-HD.

A previous systematic review [22] of randomized clinical trials aimed to understand the effectiveness of biofeedback systems on improving clinical outcomes measured as quality of life, hospitalizations, and mortality.

Several clinical trials have been undertaken since this previous systematic review. Therefore, we conducted our systematic review and meta-analysis of randomized controlled clinical trials to establish whether blood volume tracking-HD compared to C-HD is safe and effective in reducing the occurrence of intradialytic hypotension. Moreover, we attempted to identify whether the blood volume tracking system results in different clinical benefits compared to the target patient and whether it is more powerful with single or multiple controlling parameters.

## Methods

### Protocol registration

Our review adhered to a pre-specified protocol and analytical plan. The protocol was registered with the PROSPERO International Prospective Register of Systematic Reviews (registration number: CRD42023426328).

### Search strategy

The search method was designed to identify the maximum cluster of studies published from 1990 to 2022 in English. Search terms and logic are reported in the Appendix.

The search was carried out independently by two authors based on a predefined protocol set-up.

We used Medline, Cochrane library as the main electronic database and direct search of the following main journals: Journal of the American Society of Nephrology, Kidney International, Nephrology Dialysis and Transplantation, American Journal of Kidney Disease, Nephron and Clinical Nephrology, Blood Purification, International Journal of Artificial Organs.

Inclusion criteria were prospective randomized controlled trials of any experimental design (i.e., cross-over, parallel groups, etc.) which included comparisons of the incidence of intra-dialytic hypotension and blood pressure behaviour during periods of C-HD treatments versus periods of blood volume tracking-HD.

Exclusion criteria were studies which combined confounding factors like hemodiafiltration or cool dialysate in the intervention, studies on different biofeedback systems like temperature or plasma sodium, exploratory studies with fewer than 8 participants, studies on paediatric patients or patients with acute renal failure. Finally, we excluded Abstracts or congress proceedings.

### Study selection

Study selection was carried out independently by two investigators by screening all the titles and abstracts according to the inclusion and exclusion criteria described above.

### Data extraction

Data were independently extracted by two investigators according to a predefined list of data recordings: number of analyzed patients, hemodialysis schedules, length of study follow-up, number of dialysis sessions complicated by intradialytic hypotension or mean hypotension events per period. Data concerning other symptoms (e.g., cramps, nausea, vomiting, etc.), pre-dialysis, post-dialysis and change in systolic blood pressure, total ultrafiltration volume, post-dialysis body weight, post-dialysis plasma sodium concentration, and quality of life were also retrieved.

When the studies did not report the results homogeneously, we combined dichotomous and continuous data about the frequency of intradialytic hypotension, converting all the data into log odds ratio according to Chinn [23] estimates.

Whenever some statistics were not available from the papers, these were estimated by their confidence intervals and p values, and assuming the same variance. Some calculated parameters, such as changes in post dialysis systolic blood pressure or in systolic blood pressure during treatment were also estimated from the available data assuming a correlation coefficient for the calculated standard deviation equal to 0.5 [24].

When results were reported for multiple periods, the mean average and the weighted standard deviations were calculated by combining the periods and assuming a correlation equal to 0.5. Further missing statistics on primary outcome measures were obtained by interviewing the manuscript’s authors.

### Risk of bias in individual studies

Critical appraisal of the included studies was performed based on the risk of bias tool according to the Cochrane Statistical Methods Group [25].

### Summary measures and synthesis of the results

Primary outcomes were (a) dialysis complicated by hypotension; (b) intradialysis symptoms. Composite outcomes were not calculated due to the small number of studies.

Secondary safety outcomes were (a) pre-dialysis systolic blood pressure; (b) post-dialysis body weight; (c) post-dialysis plasma sodium concentration.

Event rates are described as the Odds Ratio while continuous variables are described as mean and standard deviation.

### Statistical analysis

Meta-analysis was carried out using RevMan 5.4. Effectiveness was measured by odds ratio for dialysis complicated by hypotension, and by the weighed mean difference for systolic blood pressures. The statistical method was based on the inverse variance with random effects model. We chose the random model according to the target population selected in the different studies (hypotension-prone, hypertensive or fluid overloaded), the difference in the experimental design (cross-over and parallel group design), and the length of the trials because the studies are functionally different and cannot share a common effect size.

The results of the meta-analysis refer to the overall number of treatments reported in each individual study.

To explain heterogeneity, the target population (hypotension-prone and hypertensive or fluid overloaded) and the type of biofeedback technique (single controlling variable or double controlling variable) were considered for the subgroup analysis.

Heterogeneity among the studies was assessed by the *I*^2^ statistics and a null hypothesis test, in which *p* < 0.01 indicates the presence of significant outcome heterogeneity. We assumed values of *I*^2^ between 0 and 60% as not relevant, between 60 and 80% as “substantial” heterogeneity and above 80% as “high” heterogeneity.

## Results

### Included studies and participants.

**Fig. 1:**
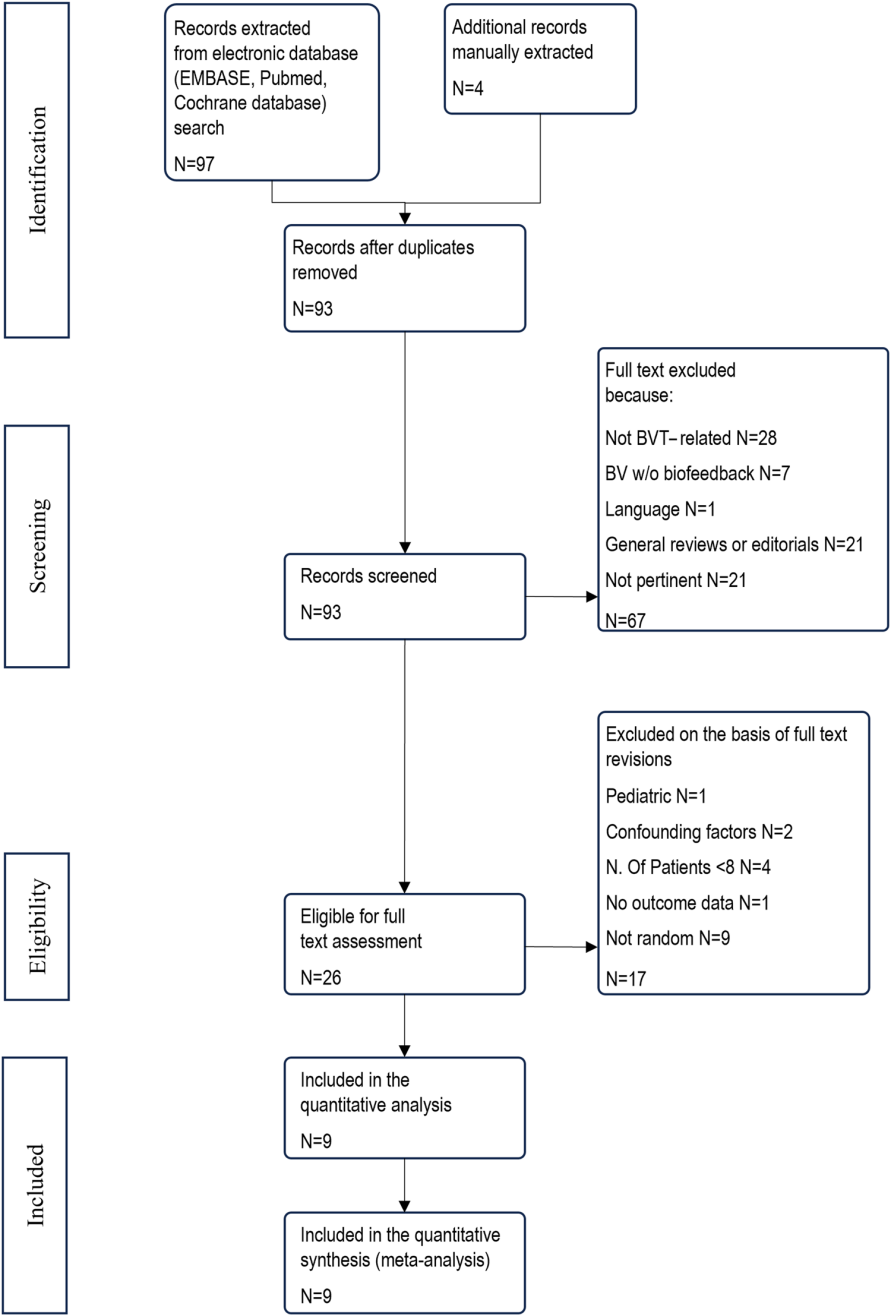
Flow diagram of literature search and study selection

We identified a total of 97 papers by using the above-mentioned search engine. Application of the inclusion and exclusion criteria yielded a final enrolment of 9 studies in the meta-analysis. Figure 1 shows the flow diagram of the study selection procedure [26–34], while Table 1 shows the main characteristics.

**Table 1:** Summary of clinical trials on the automatic blood-volume controlled hemodialysis included in the analysis

Authors	Patients & sample size	Study design	Outcomes	*P*
Ronco et al. (2000) [26] Hemocontrol–Baxter	Hypotension-prone *n* = 12	Cross-over C-HD (2 wks)–BVT (2 wks) BVT (2 wks)–C-HD(2 wks)	IDH: BVT 33% vs C-HD 82%	< 0.001
			Saline infusions ↓	< 0.001
			Rebound ↓ (eKt/V↑)	< 0.001
Santoro et al. (2002) [27] Hemocontrol–Baxter Manufacturer unrestricted grant	Hypotension-prone *n* = 36	Cross-over 2x[C-HD (4 wks)–BVT (4 wks)] 2x[BVT (4 wks)–C-HD (4 wks)]	IDH: BVT23.5% vs C-HD 33.5%	0.004
			Reduction of interdialysis symptoms	< 0.001
Moret et al. (2006) [30] Hemocontrol–Baxter	Hypotension-prone *n* = 12	Cross-over 4 phases: C-HD, BVT, SP^b^, PC^c,d^	IDH BVT 8%, C-HD 16%, Plasma conductivity controlled 14%, Sodium profile 17%	n.s
Dasselaar et al. (2007) [28] Hemocontrol–Baxter Manufacturer unrestricted grant	Hypertensive *n* = 28	Parallel groups 14 C-HD (12 wks) 14 BVT (12 wks)	IDH: BVT 0.25 ± 0.25vsC-HD 0.75 ± 0.8	0.05
			↓pre-HD and post-HD extracellular body water	0.001
			↓Cardiothoracic ratio	0.01
			No change in Brain Natriuretic Peptide	0.12
Déziel et al. (2007) [29] Hemocontrol–Baxter	Hypertensive *n* = 36	Parallel Groups C-HD (4 wks^a^ + 6 mo) BVT (4 wks^a^ + 6 mo)	Systolic and diastolic BP ↓	n.s
			Intradialytic interventions ↓	n.s
			QoL/KDQOL-SF (burden of kidney disease) ↑	0.04
			IDH: BVT27.4% vs 27.6% C-HD	0.004
Nesrallah et al. (2008) [31] Hemocontrol–Baxter Manufacturer funded	Unselected *n* = 60	Parallel Groups C-HD (4 wks^a^ + 6 mo) BVT (4 wks^a^ + 6 mo)	IDH: BVT 0.19 ± 0.46 vs C-HD 0.11 ± 0.30	< 0.01
			No change in extracellular fluid volume	n.s
			No change in QoL/dialysis-related symptoms quest	n.s
Gabrielli et al. (2009) [32] BVM-Fresenius Manufacturer funded	hypotension-prone *n* = 26	Cross-over C-HD (6 wks)–BVT (6 wks) BVT (6 wks)–C-HD (6 wks)	IDH: BVT 32.0 ± 25.5% vs CHD 40.1% ± 27.3%	0.04
			No change of SBP	n.s
Antlanger et al. (2017) [33] UCR-Nikkiso Manufacturer funded	Fluid overload *N* = 50	Parallel Groups C-HD (4 wks) BVT (4 wks) BTM (4 ws)	IDH: BVT + Temperature contr. 21% ± 21%, BVT 39% ± 27%, C-HD 34% ± 20%	0.033
			↓ Dialysis complicated by hypotensions Temperature contr. vs C-HD	0.022
			No change of BVT vs C-HD	0.93
Leung et al. (2017) [34] BVM-Fresenius Independent funding	Hypotension prone *N* = 32	Cross-over C-HD (8 wks)–BVT (8 wks) BVT (8wks)–C-HD (8 wks)	IDH: BVT 0.07 events/h vs 0.11 events/h	0.41
			Rates of asymptomatic IDH	0.64
			Proportion of HD sessions with symptomatic IDH	0.52

347 patients enrolled and 294 who completed the studies

*C-HD* conventional- hemodialysis, *BVT* Blood-Volume Tracking hemodialysis, *n.s.* not significant, *SBP* systolic blood pressure, ↓ reduced, ↑ improved

^a^Wash-out/run-in phase

^b^*SP* sodium profiling

^c^*PC* plasma conductivity-controlled feedback

^d^Eleven consecutive treatments for each modality followed by 1 week of treatment with standard dialysis

The overall number of patients enrolled in the studies was 347 and the overall number of treatments analyzed in the study was 13,274 (6481 in blood volume tracking-HD and 6793 in C-HD). Sample sizes ranged between 12 and 60 patients. Five studies used a cross-over experimental design (two were a Latin square design), while 4 used a parallel group with a two-arm design.

Five studies recruited hypotension-prone patients as the target population, two studies recruited hypertensives and two recruited fluid overloaded patients.

### Risk of bias assessment

Sequence generation was clearly described in six papers, while only four of them described the procedures for allocation concealment during randomization [28, 31, 33, 34].

Three papers stated that the intervention was single-blinded, in which the patient did not know the type of treatment delivered [27, 31, 34]. It is worth noting that personnel blindness is an unavoidable systematic error since the blood volume tracking-HD delivery is based on specific hemodialysis prescription and use of the hemodialysis monitor.

The definition of intradialytic hypotension may have resulted in reporting bias between the studies. We assumed that, whenever intradialytic hypotension was not clearly defined (but only mentioned as major intradialytic events), the sessions with major events were classified as hypotensive-complicated sessions. On the other hand, we meant that also minor events (like nausea, vomiting, yawning, cramps, etc.) could be hypotensive symptoms, so we used the number of dialysis sessions complicated by intradialytic hypotension or at least one minor event as outcome measures.

### Primary outcomes

**Fig. 2:**
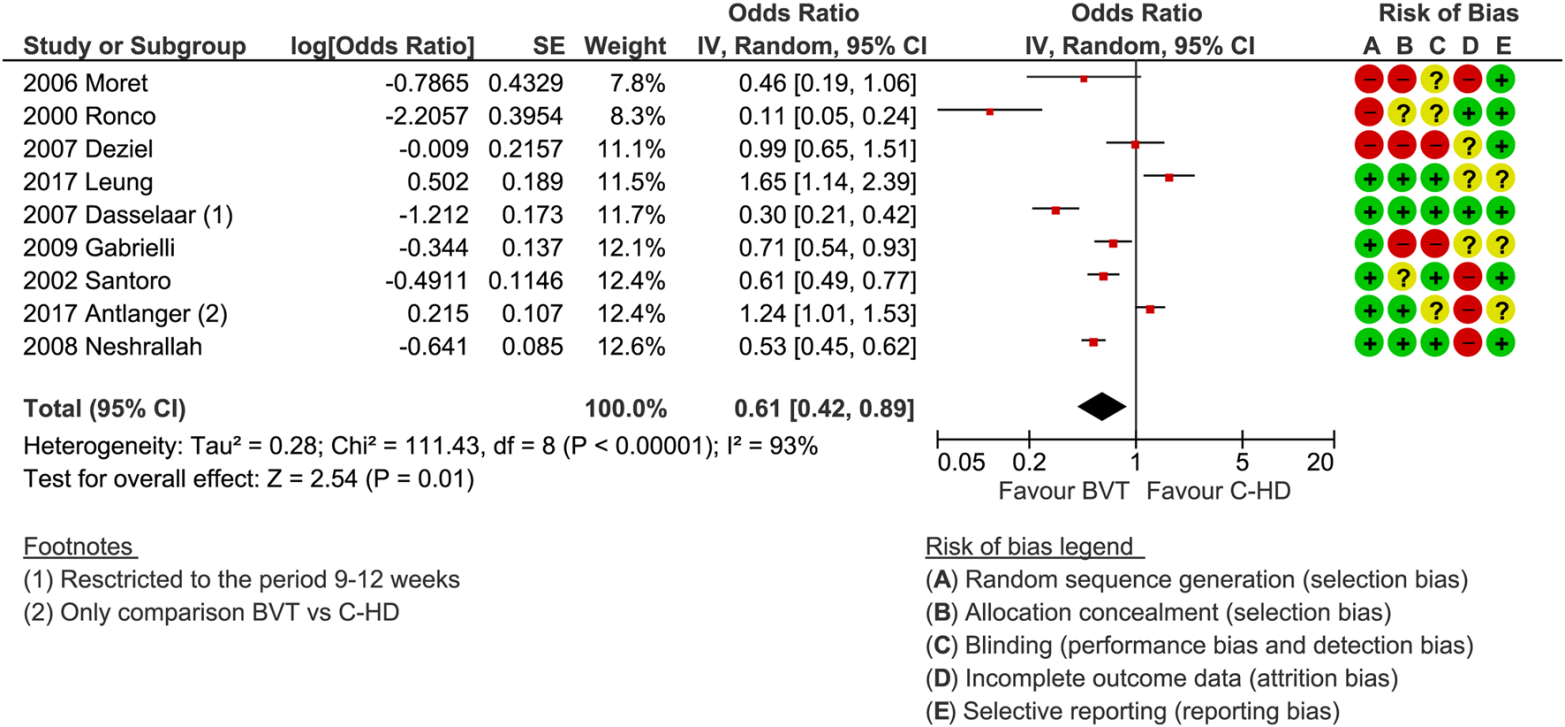
Odds ratio of intradialytic hypotension frequency expressed as dialysis complicated by hypotension

Five studies out of nine reported a significant reduction in the occurrence of acute intra-dialytic hypotension. The overall effect of blood volume tracking-HD on intra-dialytic hypotension clearly shows a reduction in complicated dialyses (Fig. 2). The odds ratio is nearly 0.61 (*p* = 0.01). Data on intra-dialytic minor events or symptoms show a significant reduction, albeit limited to two studies (odds ratio equal to 0.42%, *p* = 0.04), favouring blood volume tracking-HD as compared to C-HD.

### Secondary safety outcomes

**Fig. 3:**
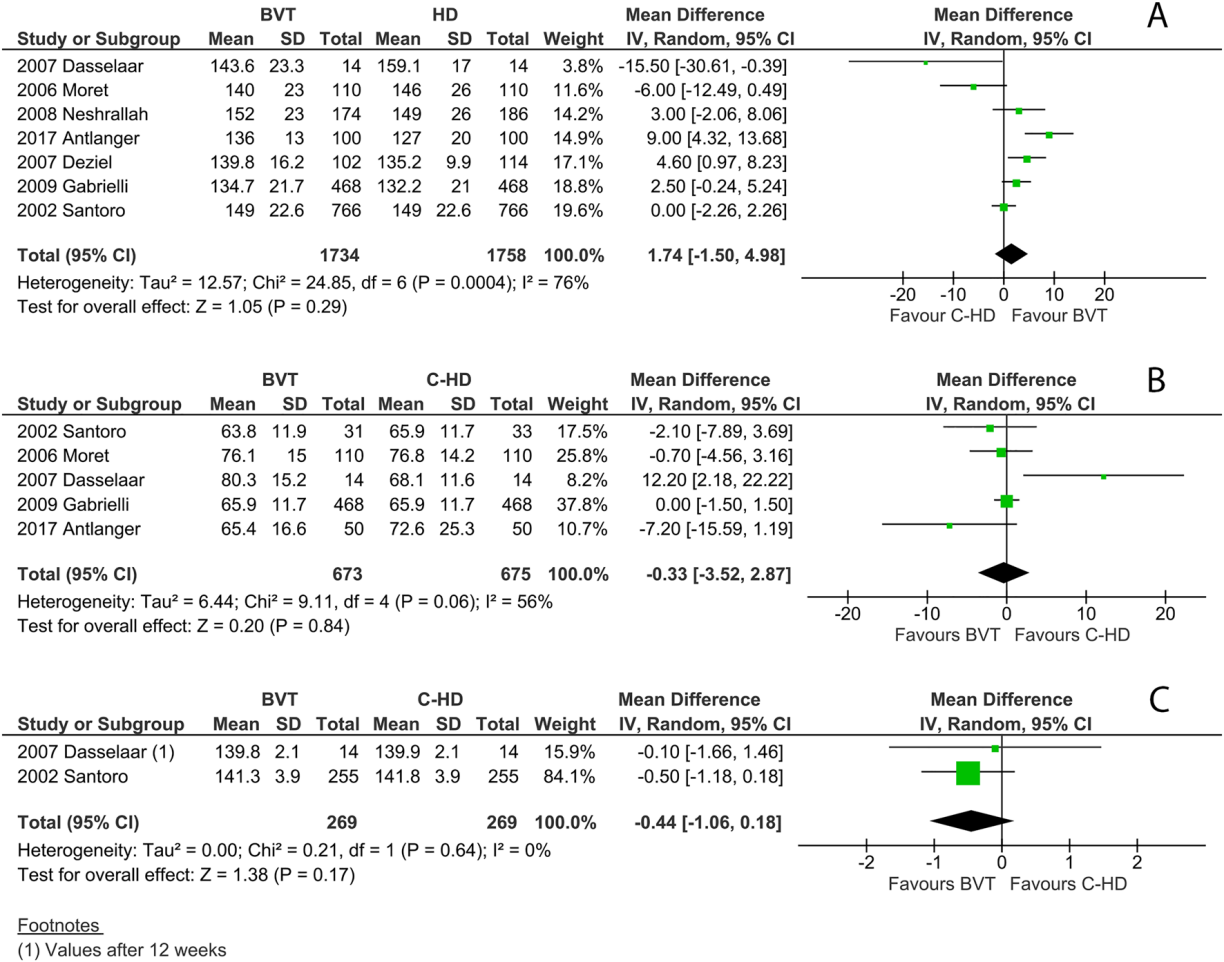
**A** pre-dialysis systolic blood pressure (mmHg); **B** post-dialysis weight (Kg); **C** post-dialysis natraemia (mMol/L)

Predialysis systolic blood pressure did not differ between the two treatments (Fig. 3A). The weighted absolute values are equal to 140.2 mmHg in C-HD and 142.0 mmHg in blood volume tracking-HD, and the overall mean difference was 1.74 mmHg (*p* = 0.29). Post dialysis weight is 330 g lower in blood volume tracking-HD than in C-HD, though not statistically significant (*p* = 0.29, Fig. 3B). It is worth noting that this result is biased by the data from Dasselaar [28]. This study has an important selection bias since the patients in the C-HD group weighed on average 68.1 kg at baseline compared to the patients in the blood volume tracking-HD group whose post dialysis weight was on average 80.7 kg. A sensitivity analysis that was run excluding this study from the overall analysis gives a post dialysis mean difference of -0.22, which is not statistically significant (*p* = 0.77).

No change can be observed for post-dialysis plasma sodium (mean difference of 0.44 mMol/L, *p* = 0.17, Fig. 3C).

### Subgroup analyses

**Fig. 4:**
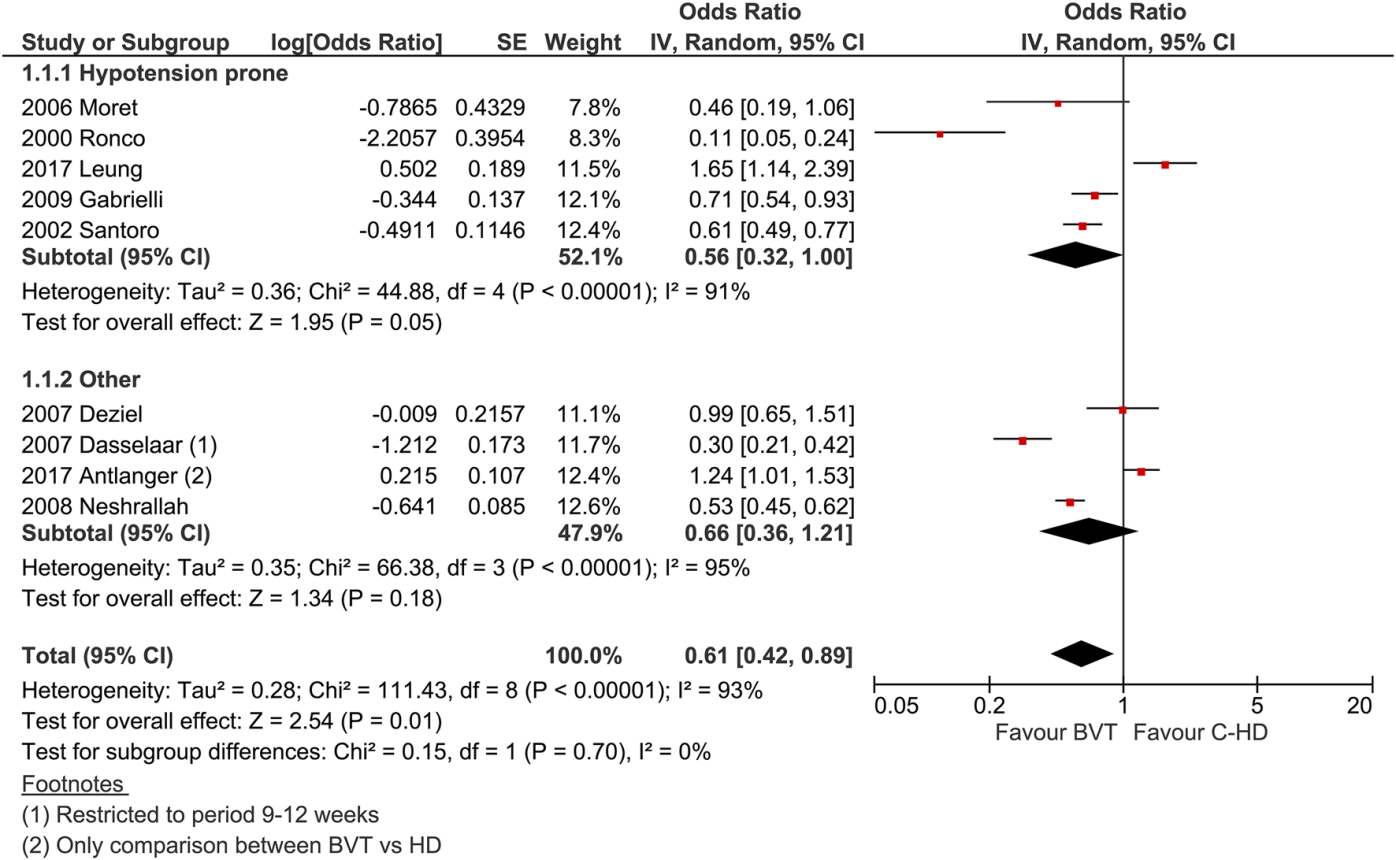
Subgroup analysis. Intradialytic hypotension frequency expressed as dialysis complicated by hypotension. Studies were grouped by target dialysis population: hypotension-prone or others which include hypertensive, fluid overloaded (Antlanger et al.) or unselected (Neshrallah et al.) patients

**Fig. 5:**
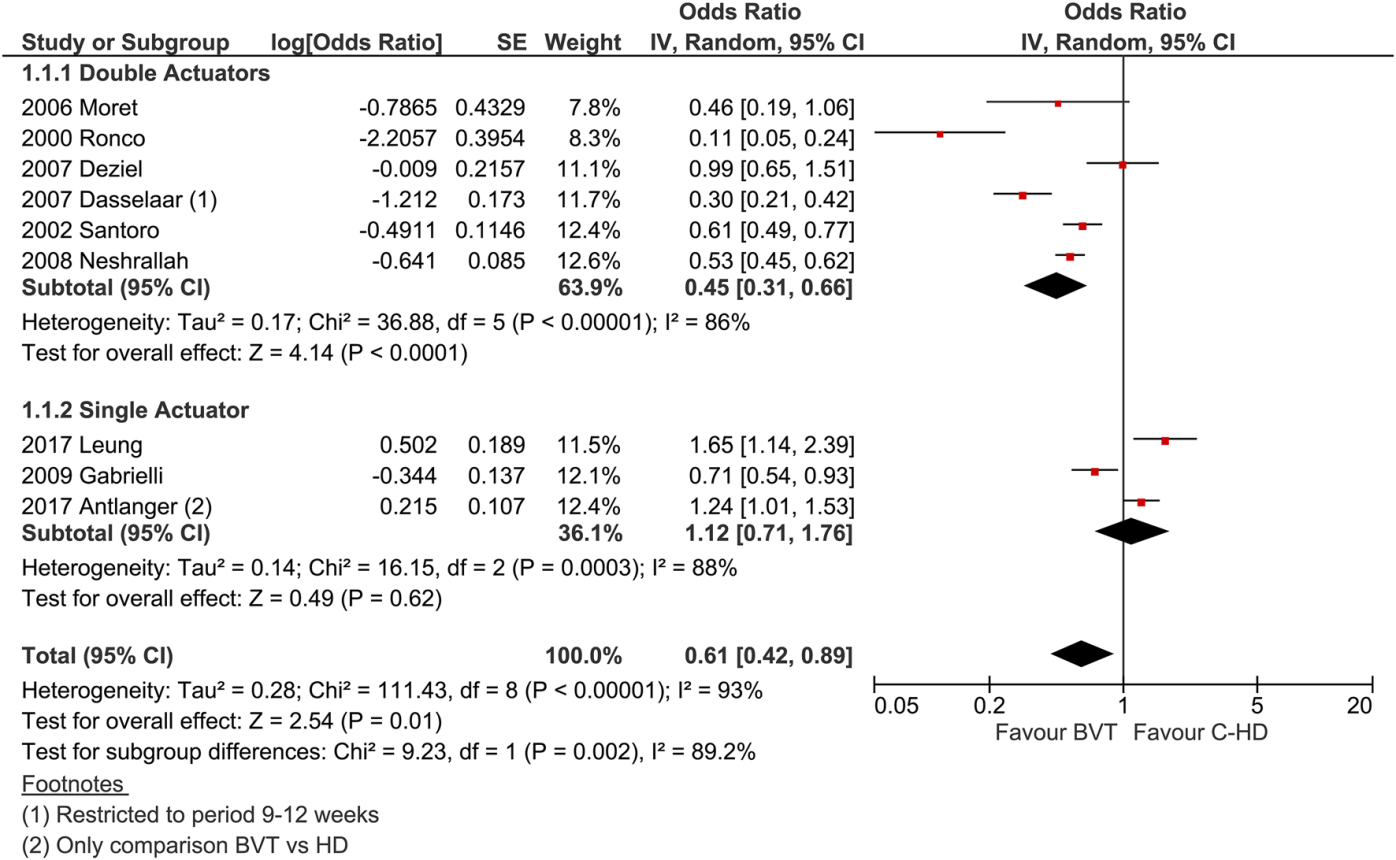
Subgroup analysis. Intradialytic hypotension frequency expressed as dialysis complicated by hypotension. Studies were grouped by type of feedback control: single controlling parameter/actuator (UR??? rate) or double (ultrafiltration rate and dialysate conductivity)

We hypothesized that both target population and type of feedback system (one or two dialysis controlling parameters) could have an impact on outcome. Indeed, by subgrouping the studies accordingly, it seems that the hypotension-prone patients benefit much more from feedback dialysis (log odds ratio 0.56, *p* = 0.05, Fig. 4), which is not the case in hypertensive or in fluid overloaded patients (odds ratio 0.66, *p* = 0.18). The effectiveness of the double parameter feedback control seems to be better than a single parameter feedback control: in fact, in the former the log odds ratio is 0.45 (*p* < 0.0001), while in the latter it is 1.12 (*p* = 0.62) as shown in Fig. 5.

## Discussion

This review shows that HD sessions with continuous control of relative blood volume changes, by means of a closed-loop system, reduce the frequency of intradialytic hypotension compared with C-HD sessions. Although different sets of studies have been included in the analysis, the main result of our meta-analysis is in line with that previously published by Neshrallah [22]. The strength of the result is stronger since the two included studies (Leung and Antlanger) show a negative effect of blood volume tracking hemodialysis. The negative effect of these studies could depend on the enrolled population (Antlanger), on the blood volume tracking technique used (Leung) and/or on the measurement of the primary end point (intradialytic hypotension).

Intradialytic hypotension remains a highly relevant problem in dialysis patients and is associated with cardiovascular mortality and all-cause mortality [4–8]. The first driver in the pathophysiology of intradialytic hypotension is the decline in blood volume due to ultrafiltration [36]. In the absence of ultrafiltration, the occurrence of intradialytic hypotension is rare [37–39]. A decline in blood volume occurs when the rate of fluid withdrawal exceeds the rate of refilling from the interstice into the vascular space. Ultrafiltration rates are related to a decline in cardiac output and to a proportional increase in intradialytic hypotension [40]. Ultrafiltration rates above 13 mL/kg/h are associated with both an increased risk of intradialytic hypotension as well as mortality [41]. On the contrary, lowering ultrafiltration rates below 13 mL/kg/h resulted in a reduction of intradialytic hypotension [43]. On the other hand, reducing ultrafiltration rate without increasing dialysis time and/or frequency leads to the risk of fluid overload and arterial hypertension in the interdialytic period. Moreover, rapid osmolar and electrolyte shifts or neurohumoral and inflammatory pathways may also affect the intradialytic cardiovascular response [41].

Relative blood volume monitoring, based on on-line monitoring of changes in hematocrit or protein, is an easy and widely used method for estimating decline in blood volume and subsequent risk of intradialytic hypotension. However, relative blood volume is a composite parameter of fluid status, plasma refilling and ultrafiltration rate [44]. Thus, interpretation in an individual patient may not be straightforward. Blood volume control is independent of individual levels and operates with a completely different logic than simple monitoring. Blood volume tracking-HD can bring the relative blood volume trends within pre-defined trajectories thus avoiding intra-dialytic ups and downs in blood volume [27]. The lower relative blood volume variability is obtained in blood volume tracking-HD by means of continuous adjustments of ultrafiltration and/or dialysate conductivity. The most intuitive advantage is the possibility to reduce the degree of hypovolemia in patients sensitive to blood volume falling, by reducing ultrafiltration when blood volume critically drops.

Sudden reductions in blood volume during the dialysis session stress the autonomic nervous system, requiring a compensatory cardiovascular response such as increases in cardiac contractility, stroke volume and peripheral vasoconstriction [42]. Continuous stress weakens the autonomic nervous system and reduces its efficiency with the obvious consequence of a less efficient cumulative response and drops in arterial blood pressure. This inadequate response is particularly evident in hypotension-prone patients, who already have a deficient cardiovascular response to hypovolemia. This is likely why, in hypotension-prone patients, the advantage of intradialytic hypotension reduction with blood volume tracking control is more evident. Furthermore, blood volume tracking-HD has another prerogative. During the periods of relative stability of blood pressure and good cardiovascular reserve, blood volume tracking increases ultrafiltration in order to recover the intradialytic weight lost during the decreased ultrafiltration periods. Studies comparing blood volume tracking-HD and C-HD reported no differences in either dry body weight or pre-dialysis blood pressure that are representative of unsatisfactory hydration control [27]. When ultrafiltration is increased, the increment in the dialysate conductivity favors plasma refilling. So, the presence of the two actuators (ultrafiltration and conductivity) in the same closed-loop control system allows to obtain a reduction in intradialytic hypotension episodes. Moreover, the blood volume tracking system maintains good control of the conductivity and thus of sodium balance [27]. Even if only a few studies report post-dialysis natremia, no increase in natremia was observed at the end of dialysis treatment [45]. 

Heterogeneity analysis can help to understand the robustness and limitation of this meta-analysis. It must be highlighted that the different target populations enrolled in the studies, the definition of the end points, the design of the studies and the different mechanisms of action of the several biofeedback systems unavoidably lead to a certain extent of heterogeneity. Since some sources of heterogeneity are known, we conducted a subgroup analysis to see how much these sources can explain the variability of the results. We limited the analysis to the target population enrolled and to the different biofeedback systems, since the end point definition (hypotension) or study design were too different across the studies and thus limited any subgrouping.

The first analysis on the target population shows that intradialytic hypotension reduction is more pronounced in the hypotension-prone patients than in the other group (fluid overloaded and hypertensive patients have been clustered together). The overall high heterogeneity (*I*^2^ = 92%) seems to be induced by the within-group dispersion of the hypotension-prone studies (*I*^2^ = 93% in the hypotension-prone group vs 79% in the other), while the between-group heterogeneity is equal to 0%. In this regard, the intradialytic hypotension reduction seems to be consistently shared by the two subgroups. Sensitivity analysis of these two groups, eliminating the outliers in the hypotension-prone studies (Leung and Ronco, where the CI’s are outside the pooled effect CI), shows that the effect size still remains unchanged (from 0.54 to 0.59) with the heterogeneity dropping to 0%. The overall results ranged from 0.63 to 0.68 within and between groups, with heterogeneity increasing from 0% to 9.9%. The underlying effect of the biofeedback system on intradialytic hypotension seems to be robust as outlined by the sensitivity.

On the other hand, if we omit the study by Dasselaar [28] in the other group, the pooled effect changes drastically from 0.79 to 1.16, thus reducing the within-group heterogeneity (*I*^2^ from 79 to 13%) but substantially increasing the between-group dispersion (*I*^2^ from 0% to 81.9%). The overall effect size indeed does not change to a great extent (from 0.63 to 0.69) even though its significance is lost (p value from 0.03 to 0.08). In conclusion, the overall intradialytic hypotension reduction in the overhydrated or hypertensive patients group seems to be affected by the presence of the study of Dasselaar, therefore highlighting that further studies are required to understand the true effect of the biofeedback system. It must be pointed out that the procedures of dry weight reduction in these study sub-groups are not homogenenous and this can impact the overall results. In fact, any intervention aimed at reducing the patients’ dry weight tends to increase the risk of an adverse event as the patient approaches the ideal dry weight.

The second subgroup analysis was carried out to determine the relative effect induced by the biofeedback system that was used. The studies were selected according to two different biofeedback algorithms driving the blood volume reduction: one with two parameters (ultrafiltration rate and dialysate conductivity), the other with one single parameter (ultrafiltration rate alone). In this case, both within-group and between-group variability is significant (*I*^2^ equal to 82% and 95% in the double actuator and single actuator, respectively, and 77% between the two groups). By omitting the study from Ronco, the sensitivity analysis again changes the variability within the double actuator group (*I*^2^ from 82 to 54%), but not the overall heterogeneity (*I*^2^ from 92 to 90%). In conclusion, the effect of hypotension reduction seems to be robust and significant within the double actuator group.

A further effect on heterogeneity could be due to the different definition of the endpoint of intradialytic hypotension. Subgroup analysis cannot be carried out since each study adopted a different definition of intradialytic hypotension, as outlined in Table 2. A more homogeneous consensus around this definition could help to compare the true effect of different hemodialysis techniques on this dialysis side effect.

**Table 2:** Definition of intradialytic hypotension of the included studies

Author	Definition
Ronco et al. (2000) [26]	ΔSBP > 40 mmHg respect to pre-dialysis SBP w/o symptoms Or ΔSBP < 40 mmHg respect to pre-dialysis SBP with symptoms
Santoro et al. (2002) [27]	SBP ≤ 90 mmHg w/o symptoms if predialysis SBP ≥ 100 mmHg Or ΔSBP > 10% of predialysis SBP with symptoms Or ΔSBP > 25 mmHG disregarding predialysis SBP
Moret et al. (2006) [30]	SBP < 100 mmHg if predialysis SBP < 110 mmHg Or ΔSBP > 30 mmHg with symptoms and need of intervention
Dasselaar et al. (2007) [28]	ΔSBP > 40 mmHg respect to predialysis SBP requiring intervention
Déziel et al. (2007) [29]	Not specified but symptoms requiring nurse intervention (Trendelenburg, UF reduction or blood flow, saline infusion)
Nesrallah et al. (2008) [31]	ΔSBP > 10 mmHg requiring intervention
Gabrielli et al. (2009) [32]	Any SBP drop with symptoms and requiring intervention
Antlanger et al. (2017) [33]	ΔSBP > 40 mmHg within 30 min w/o symptoms Or ΔSBP > 40 mmHg within 30 min w symptoms Or Un specific drop of SBP with symptoms
Leung et al. (2017) [34]	ΔSBP > 20 mmHg with symptoms

This analysis has several strengths. First, it clarifies the difference between blood volume monitoring and blood volume control where the latter is a proactive system that mimics the action of the clinician who adapts ultrafiltration and dialysate conductivity to the intra-dialysis behaviour of the blood volume changes. Secondly, we can conclude that the biofeedback systems are particularly beneficial for hypotension-prone patients, and that the effect is enhanced by the combined use of several control systems affecting blood pressure. The potential benefit on the reduction of dry weight needs further investigation to reach conclusive results.

## Conclusions

The use of systems controlling blood volume trends during HD via a closed loop may be effective in reducing the incidence of intradialytic hypotension and making it easier to achieve the patients’ ideal dry body weight. Both clinical objectives may have a greater influence on patient outcome. In fact, hypotensive episodes during HD are associated with worsened patient survival. On the other hand, failure to achieve the patients’ ideal dry body weight can lead to chronic fluid overload that is responsible for arterial hypertension and deleterious effects on the cardiovascular system. However, new studies on chronic HD patients examining the long-term effects of the use of blood volume tracking systems on real hard endpoints, such as patient survival and the risk of cardiovascular events in chronic HD patients, are needed.

## Search method

### Medical headings search terms

#### H(a)emodialysis

(((("haemodialysis"[All Fields] OR "renal dialysis"[MeSH Terms] OR ("renal"[All Fields] AND "dialysis"[All Fields]) OR "renal dialysis"[All Fields] OR "hemodialysis"[All Fields]) OR ("haemodialysis"[All Fields] OR "renal dialysis"[MeSH Terms] OR ("renal"[All Fields] AND "dialysis"[All Fields]) OR "renal dialysis"[All Fields] OR "hemodialysis"[All Fields])) AND ("blood volume tracking"[All Fields] OR hemocontrol[All Fields] OR "blood temperature"[All Fields] OR BVM[All Fields] OR Diascan[All Fields] OR Diacontrol[All Fields] OR ("Fuzzy Logic"[All Fields] AND "blood Pressure"[All Fields]))) AND "2009/09/27 08.10"[MHDA]: "2012/06/10 16.12"[MHDA]) NOT (((("haemodialysis"[TIAB] OR "renal dialysis"[TIAB] OR ("renal"[TIAB] AND "dialysis"[TIAB]) OR "renal dialysis"[TIAB] OR "hemodialysis"[TIAB]) OR ("haemodialysis"[TIAB] OR "renal dialysis"[TIAB] OR ("renal"[TIAB] AND "dialysis"[TIAB]) OR "renal dialysis"[TIAB] OR "hemodialysis"[TIAB])) AND ("blood volume tracking"[TIAB] OR hemocontrol[TIAB] OR "blood temperature"[TIAB] OR BVM[TIAB] OR Diascan[TIAB] OR Diacontrol[TIAB] OR ("Fuzzy Logic"[TIAB] AND "blood Pressure"[TIAB]))) AND "0001"[EDAT]: "2009/09/27 08.10"[EDAT]).
